# An Atypical Presentation of Myasthenia Gravis: A Case Report

**DOI:** 10.7759/cureus.4563

**Published:** 2019-04-29

**Authors:** Hannan Asghar, Fahad N Sheikh, Heena Dev, Milenko B Lazarevic, Syed Adeel Hassan

**Affiliations:** 1 Internal Medicine, Sahiwal Medical College, Sahiwal, PAK; 2 Medicine, Sahiwal Medical College, Sahiwal, PAK; 3 Internal Medicine, Windsor University School of Medicine, Cayon, KNA; 4 Internal Medicine, Swedish Covenant Hospital, Chicago, USA; 5 Internal Medicine, Dow University of Health Sciences, Karachi, PAK

**Keywords:** neck, weakness, atypical, elderly

## Abstract

Myasthenia gravis (MG) belongs to a spectrum of autoimmune diseases in which anti-acetylcholine receptor antibodies damage neuromuscular junctions. It is a relatively rare disease with a higher incidence among the female population. The classical presentation is fatigable fluctuating diplopia or ptosis and, uncommonly, dysphagia or dysphonia. Even though it is rare, this condition can affect any skeletal muscle groups, including the neck or proximal limb muscles. There have been no reported cases of MG presenting as isolated neck weakness. An 81-year-old female patient presented with neck weakness associated with mild discomfort that progressively worsened throughout the day. Examination revealed reduced cervical muscular motor strength only. All imaging and laboratory investigations were within normal limits, except anti-acetylcholine receptor antibodies (binding Ab 12.04 nmol/L, blocking Ab 52% while modulating Ab 84%) with moderately elevated creatine phosphokinase (CPK) levels (350 U/l). The patient was prescribed Mestinon 60 mg QID (pyridostigmine), which led to rapid and significant relief of neck weakness. The patient has been stable on the medication for two years. MG typically presents in middle-aged female populations but, rarely, can also present with atypical symptoms among the elderly. Clinicians should have a high index of suspicion for myasthenia presenting with fatigable muscle weakness to reduce investigative costs and morbidity.

## Introduction

Myasthenia gravis (MG) is an established autoimmune disease commonly affecting middle-aged female populations [1]. This disease is mediated by a type-II antibody reaction in which antibodies directed against post-synaptic nicotinic acetylcholine receptors attack the myoneural junction and damage the post-synaptic membrane via complement fixation. This results in the failure of action potential propagation across the neurons, eventually leading to a neuromuscular weakness without stiffness [2]. Classically, the anticholinergic autoantibodies target the extraocular muscles, leading to fluctuating muscular fatigability, predominantly resulting in bilateral diplopia and ptosis, which is typically worse at the end of the day. It constitutes more than half the cases [1]. Bulbar weakness, leading to dysphagia and dysarthria, has been rarely described as the initial complaint, more commonly in the elderly male population [2].

## Case presentation

The clinical case we report here is of an 81-year-old female with a significant past medical history of diabetes, hypertension, and degenerative joint disease of the right hip joint. She reported neck weakness for two weeks, which she described as an inability to hold the neck straight. It began without any preceding trauma, infection, or significant stressor. Neck weakness started suddenly, worsening throughout as the day progresses, causing mild pain with no notable aggravating or alleviating factors. Staff at the nursing home noticed that the patient was having difficulty in extending her neck. There appeared to be no diplopia, ptosis, dysphagia, dysarthria, dysphonia, regurgitation, neck stiffness, photophobia, fever/chills, or difficulty breathing. There is no significant family history of autoimmune diseases except for hypothyroidism in the mother.

Detailed examination revealed 3/5 strength of the neck musculature but intact strength in the upper and lower extremities. Spurling and Lhermitte’s signs were negative. No sensory impairment was noticed. Deep tendon reflexes were 2+ symmetric all over, with Babinski negative. The differential diagnosis at this point included degenerative joint disease, vertebral compression fracture, muscular dystrophy, neuromuscular disease, and paraneoplastic process. Initial blood tests were within normal limits. The patient underwent a cervical spine X-ray and blood tests for inflammatory markers and metabolic panel. The X-ray was unremarkable, and there were no signs of inflammation or infection on complete blood count (CBC) and comprehensive metabolic panel (CMP). Liver function tests (LFTs), including aspartate transaminase (AST), alanine transaminase (ALT), and alkaline phosphatase (ALP), were within normal limits, excluding muscular inflammation. Autoimmune markers came out normal as well. The erythrocyte sedimentation rate (ESR) was 15 mm/hr, and the serum creatine phosphokinase (CPK) levels were mildly elevated at 350 U/l (normal 22-198 U/l).

The patient presented two weeks later with a progression in neck weakness and difficulty in holding her neck still. Chest X-ray was unremarkable, with no signs of thymoma. The anti-acetylcholine receptor binding antibody was 12.04 nmol/L (normal <0.40 nmol/L), blocking antibody was 52% (normal <26%), and modulating antibody was 84% (normal <45%), which are consistent with a diagnosis of myasthenia gravis. Depending on the clinical picture and laboratory results, we decided to prescribe cholinergic agonists rather than performing electromyography to diagnose myasthenia.

The patient was introduced on pyridostigmine (Mestinon 60 mg QID) therapy. We advised her to follow up in three days. Her symptoms improved drastically following the commencement of pyridostigmine therapy. The disease was not aggressive in course. The patient is stable on the medication since then, with no recurrence of weakness. There have been no reported adverse effects of therapy. The patient returned to her previous baseline lifestyle without any troubles with vision, swallowing, speech, or gait.

## Discussion

The case described above is an extremely rare presentation of a rare condition, even though myasthenia gravis is the most common neuromuscular junction disorder. This case highlights the importance of a high clinical index of suspicion for myasthenia in elderly patients, with unexplainable neck or bulbar weakness even when there is no classical fluctuating weakness. Weakness in myasthenia is due to impaired action potential propagation caused by damage to post-synaptic acetylcholine receptors due to which muscles do not depolarize. The annual incidence of MG is relatively less, often 10-20 newly reported cases per million [3]. The disease follows a bimodal pattern of distribution, with a peak in the second to third decades among the female population and another peak in the fourth to eighth decades among the male population [4].

Classically, it presents with fluctuating and fatigable skeletal muscle weakness, commonly affecting the extraocular muscles and the muscles of mastication to a lesser extent. There have been reports of myasthenia presenting as dysphagia in elderly males with rapid progression to respiratory failure [2]. Apart from prototypical muscular involvement, any set of muscles can be affected, including the proximal limb and neck musculature.

Significant delays in diagnoses or frequent misdiagnoses among elderly patients have been reported [5]. The estimated time for diagnosis in the elderly (>60 years) was 4.5 months in juxtaposition to 2.5 months in the younger age groups [5]. Among elderly females, neck weakness could be a manifestation of degenerative joint disease, disk herniation, osteoporotic vertebral fractures, and metastases. Owing to a vast amount of possible causes for neuromuscular symptoms, including transient ischemic attack (TIA) or stroke, Parkinson’s disease, motor neuron disease, neuropathies, and Horner syndrome, myasthenia is thought to be under-diagnosed in senile patients [6].

Traditional treatment for stable myasthenia gravis is the use of indirect acting anticholinesterases like pyridostigmine/neostigmine [7]. Ample therapy with anticholinesterases and steroids directly lead to the desired survival rate in elderly populations [8]. Thymectomy is an appropriate option to control symptoms in younger patients, but it is not typically favored in elderly patients, especially those above 60 years old [6]. Elderly patients have often shown a good response to therapies in general [7].

## Conclusions

There have been no reported cases of myasthenia presenting only with neck weakness in the elderly female population. The elderly are at risk of misdiagnosis, leading to further complications. To sum up, this case shows that myasthenia should be under consideration as a possibility in elderly patients presenting with neck weakness despite the absence of classical symptoms. It is important to diagnose myasthenia in its early stages and manage it appropriately.
